# Cyanescent *Gyroporus* (Gyroporaceae, Boletales) from China

**DOI:** 10.3897/mycokeys.81.65660

**Published:** 2021-07-26

**Authors:** Cong Huang, Ming Zhang, Xing-Liang Wu, Gang Wu, Jian-Ping Xu, Zhu L. Yang, Yan-Chun Li

**Affiliations:** 1 Key Laboratory for Plant Diversity and Biogeography of East Asia, Kunming Institute of Botany, Chinese Academy of Sciences, 650201, Kunming, China Kunming Institute of Botany, Chinese Academy of Sciences Kunming China; 2 Yunnan Key Laboratory for Fungal Diversity and Green Development, Kunming 650201, Yunnan, China University of Chinese Academy of Sciences Beijing China; 3 University of Chinese Academy of Sciences, Beijing 100049, China Institute of Microbiology, Guangdong Academy of Sciences Kunming China; 4 Institute of Microbiology, Guangdong Academy of Sciences, Guangzhou 510070, China Hainan University Hainan China; 5 College of Tropical Crops, Hainan University, Hainan 570208, China Yunnan Key Laboratory for Fungal Diversity and Green Development Kunming China; 6 Department of Biology, McMaster University, Hamilton, ON L8S 4K1, Canada McMaster University Hamilton Canada

**Keywords:** Boletes, distribution, new taxa, phylogeny, taxonomy

## Abstract

*Gyroporus* species with cyanescent oxidation reactions were investigated, based on morphology and phylogenetic analysis of DNA sequences from the nuclear ribosomal large subunit (nrLSU), the nuclear ribosomal internal transcribed spacer (ITS) and the mitochondrial adenosine triphosphate ATP synthase subunit 6 (*atp6*). Three species, including two new species, namely *G. alpinus* and *G. flavocyanescens* and one previously-described species, namely *G. brunneofloccosus*, are revealed from China. Collections formerly reported from China as “*G. cyanescens*” are either *G. alpinus* or *G. flavocyanescens*. The new species are documented and illustrated in detail, while the concept of *G. brunneofloccosus* is refined with additional recently-collected materials. Additionally, the cyanescent species *G. pseudomicrosporus*, previously described from China, is shown to be a member of the genus *Gyrodon*, based on re-examination of the type specimen. A key to the cyanescent *Gyroporus* species from China is provided.

## Introduction

The genus, *Gyroporus* Quél., typified by *G. cyanescens* (Bull.) Quél., is a boletoid genus in the monogeneric family Gyroporaceae in the suborder Sclerodermatineae (Boletales) (Binder & Bresinsky, 2002). More than 40 species have been reported and described in this genus (e.g. Li et al. 2003; Kirk et al. 2008; Vizzini et al. 2015; Crous et al. 2016, 2017; Das et al. 2017; Davoodian et al. 2018, 2019, 2020; Magnago et al. 2018). *Gyroporus* is characterised by the initially spongy and then hollow stipe, the white to cream to yellow hymenophore, the white to yellowish context without colour change or with cyanescent or brownish colour change when bruised, the ellipsoid to broadly ellipsoid basidiospores and the presence of clamp connections (Singer 1986; Watling 2008; Magnago et al. 2018). Globally, so far fifteen species have been reported with cyanescent colour changes when bruised. Amongst these, eight species were reported from the Northern Hemisphere: four species originally described from Europe (*G. cyanescens*, *G. lacteus* Quél., *G. pseudolacteus* G. Moreno, Carlavilla, Heykoop, Manjón & Vizzini and *G. pseudocyanescens* G. Moreno, Carlavilla, Heykoop, Manjón & Vizzini), two species originally described from East Asia (*G. brunneofloccosus* T.H. Li, W.Q. Deng & B. Song and *G. pseudomicrosporus* M. Zang), one species originally described from North America [*G. violaceotinctus* (Watling) Blanco-Dios] and one species originally described from Central America (*G. phaeocyanescens* Singer & M.H. Ivory) (Bulliard 1788; Léveillé 1848; Watling 1969; Singer et al. 1983; Zang 1986; Li et al. 2003; Vizzini et al. 2015; Crous et al. 2016, 2017). Seven cyanescent species were reported from Australia (Southern Hemisphere), including four validly-published species (*G. australiensis* Davoodian, N.A. Fechner & Halling, *G. furvescens* Davoodian & Halling, *G. occidentalis* Davoodian, Bougher & Halling and *G. robinsonii* Davoodian) and three undescribed species proposed by Davoodian (2018) with provisional names (*G. allocyanescens*, *G. austrocyanescens* and *G. neocyanescens*) which need additional study when more collections are acquired (Davoodian et al. 2019, 2020). In China, three cyanescent *Gyroporus* have been reported: *G. cyanescens*, *G. brunneofloccosus* and *G. pseudomicrosporus* (Zang 1986; Li et al. 2003). During our recent field investigations of *Gyroporus* across China, we encountered two impressive cyanescent species from south-western China which are apparently different from other species in this genus.

In this study, we used both morphological data and molecular sequences from the nuclear ribosomal large subunit (nrLSU), the nuclear ribosomal internal transcribed spacer (ITS) and the mitochondrial adenosine triphosphate ATP synthase subunit 6 (*atp6*), together with ecological data to evaluate the phylogenetic relationships of the cyanescent species within *Gyroporus* and make morphological and ecological comparisons.

## Materials and methods

### Sampling and morphological studies

The collections of cyanescent species in *Gyroporus* were collected from Guizhou, Yunnan and Guangdong Provinces, China, in forests dominated by plants of the family Fagaceae or in the mixed forests dominated by plants of the families Fagaceae and Pinaceae. Fresh basidiomata were photographed and macroscopic characteristics, habitat, colour change when bruised, odour and taste were recorded. Basidiomata were then dried and deposited in the Herbarium of the Kunming Institute of Botany, Chinese Academy of Sciences (**KUN**) and the Herbarium of the Guangdong Institute of Microbiology (**GDGM**). Macroscopic descriptions and microscopic studies followed Naseer et al. (2020), Zhang et al. (2019) and references therein. Colour description was according to Kornerup and Wanscher (1981). The notations and statistics of basidiospores followed Liu et al. (2020). Line drawings were prepared by free hand.

### DNA extraction, PCR and sequencing

Genomic DNA was extracted from 100 mg of silica-gel dried samples or herbarium materials using the modified CTAB method (Doyle and Doyle 1987). PCR amplification primers ITS1 and ITS4 were used for the ITS region, LROR and LR5 were used for nrLSU and ATP6-F and ATP6-R were used for *atp6* (White et al. 1990; Davoodian et al. 2018). PCR, amplification conditions, sequencing and sequence alignment followed those in Gelardi et al. (2019), Huang et al. (2021) and Gómez-Zapata et al. (2021).

### Phylogenetic analysis

The phylogenetic analyses were based on three fragments (*atp6*, ITS and nrLSU). Two datasets, the *atp6* dataset and the combined nrLSU and ITS dataset, were analysed using RAxML (Stamatakis et al. 2008). DNA sequences of the cyanescent species of *Gyroporus* from China and other continents (Crous et al. 2016, 2017; Das et al. 2017; Davoodian et al. 2018; Magnago et al. 2018) were used to infer the phylogenetic relationships between these species. Since seven cyanescent species have been reported from the Southern Hemisphere continent of Australia and their *atp6* sequences are publicly available, the *atp6* dataset was used to infer relationships of Australian cyanescent species with those from Europe, North America and East Asia in the Northern Hemisphere. The combined dataset was mainly used to infer relationships of species from East Asia, North America and Europe. In our preliminary analysis, the cyanescent species formed a monophyletic clade, thus, *G. longicystidiatus* Nagas. & Hongo without colour change when bruised was chosen as outgroup. For the combined dataset, *Scleroderma areolatum* Ehrenb., *S. duckei* B.D.B. Silva, M.P. Martín & Baseia and *S. laeve* Lloyd were selected as outgroup taxa.

The combined dataset was partitioned into four partitions (nrLSU, ITS1, 5.8S and ITS2). Statistical support for the phylogentic analyses was determined using a rapid bootstrapping with 1000 replicates in Maximum Likelihood (ML) analysis under the partitioned GTRGAMMA model. The scientific names, collection information and GenBank accession numbers for the specimens used in the phylogenetic analyses are presented in Table 1.

**Table 1.** A tabulation of specimens used for molecular phylogenetic analyses in the present study. Sequences newly generated in this study are indicated in bold.

Species	Voucher	Locality	GenBank Accession No.		
			ITS	LSU	*atp6*
“*Gyroporus allocyanescens*”	REH9700A	Australia	–	–	MF818179
*G. alpinus*	LI1478-Strain1	China	**MW149435**	**MW151268**	**MW452609**
*G. alpinus*	LI1478-Strain2	China	**MW149438**	**MW151269**	**MW452610**
*G. ammophilus*	AH45842	Spain	KX869876	KX869890	–
*G. ammophilus*	AH45814	Spain	KX869878	KX869892	–
*G. australiensis*	REH9501	Australia	–	–	MF818183
*G. australiensis*	REH9559	Australia	–	–	MF818182
*G. austrobrasiliensis*	ACM1136	Brazil	MF436999	MF437014	–
*G. austrobrasiliensis*	ACM1144	Brazil	MF437000	MF437015	–
“*G. austrocyanescens*”	REH9700	Australia	–	–	MF818176
*G. brunneofloccosus*	GDGM74638	China	**MW149437**	**MW151266**	–
*G. brunneofloccosus*	WU2644	China	**MW149436**	**MW151267**	**MW452611**
*G. brunneofloccosus*	OR482	China	–	–	MF818146
*G. castaneus*	AH45841	Spain	KX869875	KX869889	–
*G. castaneus*	AH45844	Spain	KX869874	KX869888	–
*G. cyanescens*	MCVE17184 (epitype)	Italy	JF908785	–	–
*G. cyanescens*	2837	Canada	KM248948	–	–
*G. cyanescens*	MCVE:28580	Italy	KT363684	KT363685	–
*G. cyanescens*	MB05-04	USA	–	EU718102	–
*G. cyanescens*	MG639a	Italy	–	–	MF818172
“*G. cyanescens*”	REH9970	USA	–	–	MF818174
“*G. cyanescens*”	ND11	USA	–	–	MF818173
“*G. cyanescens*”	KH-JPN15-0733	Japan	–	–	MF818191
“*G. cyanescens*”	KH-JPN15-0745	Japan	–	–	MF818192
“*G. cyanescens*”	NY1782681	South Korea	–	–	MF818185
*G. flavocyanescens*	WXL1182	China	**MW440550**	**MW442950**	**MW452613**
*G. flavocyanescens*	WXL1187	China	**MW440551**	**MW442951**	–
*G. furvescens*	REH9673	Australia	–	–	MF818175
*G. lacteus*	MCVE28582 (epitype)	Italy	KT363682	KT363683	–
*G. longicystidiatus*	REH8799	Thailand	EU718106	EU718142	MF818147
*G. longicystidiatus*	EN99-67	Japan	–	–	MF818151
*G. occidentalis*	REH8821 (holotype)	Australia	EU718103	EU718139	MF818177
*G. occidentalis*	REH8819	Australia	–	EU718172	–
*G. occidentalis*	E8164	Australia	–	–	MF818194
*G. paramjitii*	REH8804	Thailand	EU718101	EU718137	–
*G. paramjitii*	KD 16-002	India	MF120284	MF120285	–
*G. phaeocyanescens*	ARB1309	USA	–	–	MF818144
*G. pseudocyanescens*	AH55729 (holotype)	Spain	KY576808	KY576806	–
*G. pseudocyanescens*	AH45840	Spain	KY576809	KY576807	–
*G. pseudolacteus*	AH45848	Spain	KX869867	KX869881	–
*G. pseudolacteus*	AH39364 (holotype)	Spain	KX869866	KX869880	
*G. purpurinus*	PRL3737	USA	EU718105	EU718141	–
*G. robinsonii*	ND13	Australia	–	–	MF818178
*G. robinsonii*	OKM23719	Australia	–	EU718140	–
*G. umbrinisquamosus*	BUF-Both3525	USA	–	–	MF818145
*Scleroderma areolatum*	PBM2208	–	–	EU718150	–
*S. duckei*	INPA 272127	–	NR_147664		–
*S. laeve*	ZLR46	China	MW553325	MW553729	–

## Results

### Molecular analysis

**Figure 1.**
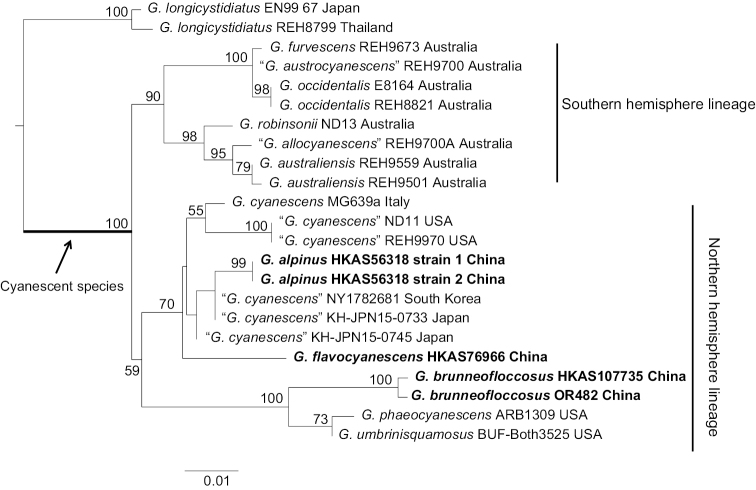
Maximum Likelihood phylogenetic tree of *Gyroporus* inferred from the *atp6* dataset. Bootstrap frequencies (> 50%) are shown above or below supported branches. Newly-sequenced collections are indicated in bold. Species vouchers and countries of origin are provided after the species name successively.

**Figure 2.**
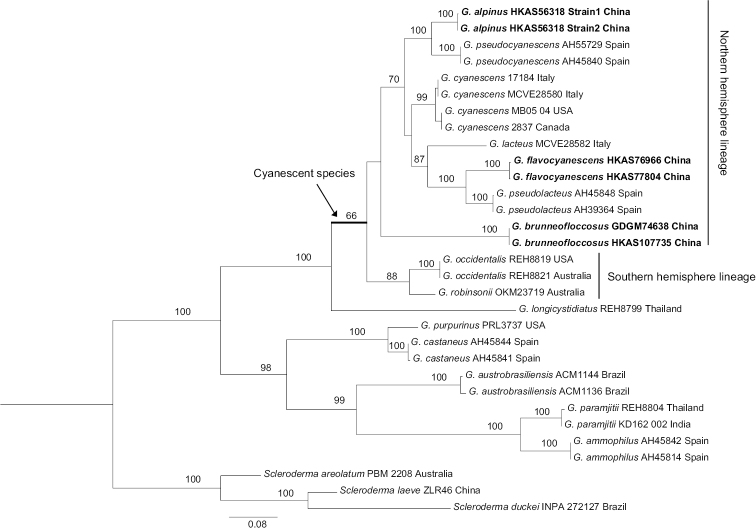
Maximum Likelihood phylogenetic tree of *Gyroporus* inferred from the combined (nrLSU + ITS) dataset. Bootstrap frequencies (> 50%) are shown above supported branches. Newly-sequenced collections are indicated in bold. Species vouchers and countries of origin are provided after the species name successively.

In this study, sixteen new sequences of *Gyroporus* (six for ITS, six for nrLSU and four for *atp6*) were generated. Two datasets were analysed: the combined nuclear ribosomal DNA dataset (nrLSU + ITS) consists of 31 sequences and is 1720 bp long; the mitochondrial *atp6* dataset consists of 23 sequences and is 596 bp long. The alignments were submitted to TreeBASE (27864). Phylograms inferred with RAxML, including the support values, are illustrated (Figs 1, 2). In both of our analyses, species with cyanescent colour changes when bruised cluster together with high support (100% in Fig. 1 and 99% in Fig. 2).

The phylogenetic analysis of *atp6* data indicates that the Australian cyanescent *Gyroporus* species form an independent lineage, while the other cyanescent species from the Northern Hemisphere form another lineage (Fig. 1). It should be noted that the Northern Hemisphere lineage has relatively low bootstrap support (59%) in the *atp6* analysis; however, its two main constituent sub-lineages have high support (70% and 100%) (Fig. 1). Three cyanescent species from China that belong to the Northern Hemisphere lineage are revealed, including two new species, namely *G. alpinus* Yan C. Li, C. Huang & Zhu L. Yang and *G. flavocyanescens* Yan C. Li, C. Huang & Zhu L. Yang, and one previously-described species, namely *G. brunneofloccosus*. The phylogenetic analysis of the combined (nrLSU + ITS) dataset also indicates that the Australian cyanescent *Gyroporus* species form an independent Southern Hemisphere lineage, while the other cyanescent species from the Northern Hemisphere form another lineage, yet without statistical bootstrap support, but its two main constituent sub-lineages also have high and moderate support (100% and 70%) (Fig. 2). The Chinese species *G. brunneofloccosus* forms one of the two well-supported (100%) sub-lineages, while the other species in Northern Hemisphere form another moderately supported (70%) sub-lineage. The newly-described species *G. alpinus* is closely related to *G. pseudocyanescens* G. Moreno, Carlavilla, Heykoop, Manjón & Vizzini, while *G. flavocyanescens* is closely related to *G. pseudolacteus* G. Moreno, Carlavilla, Heykoop, Manjón & Vizzini.

### Taxonomy

#### Gyroporus alpinus

Taxon classificationFungiBoletalesGyroporaceae

Yan C. Li, C. Huang & Zhu L. Yang sp. nov.

C57A7F97-C212-542D-A612-5ACEF2EFB4F5

838413

Figs 3a–c , 4

##### Etymology.

The epithet *alpinus* refers to its distribution in alpine forests.

##### Type.

China. Yunnan Province: Deqin, Shangri-La County, Haba Snow Mountain, Yang Fang, alt. 3800 m, 14 Aug 2008, Y.C. Li 1478 (KUN-HKAS 56318, GenBank accession numbers: MW149435 and MW149438 for ITS, MW151268 and MW151269 for nrLSU, MW452609 and MW452610 for *atp6*).

**Figure 3.**
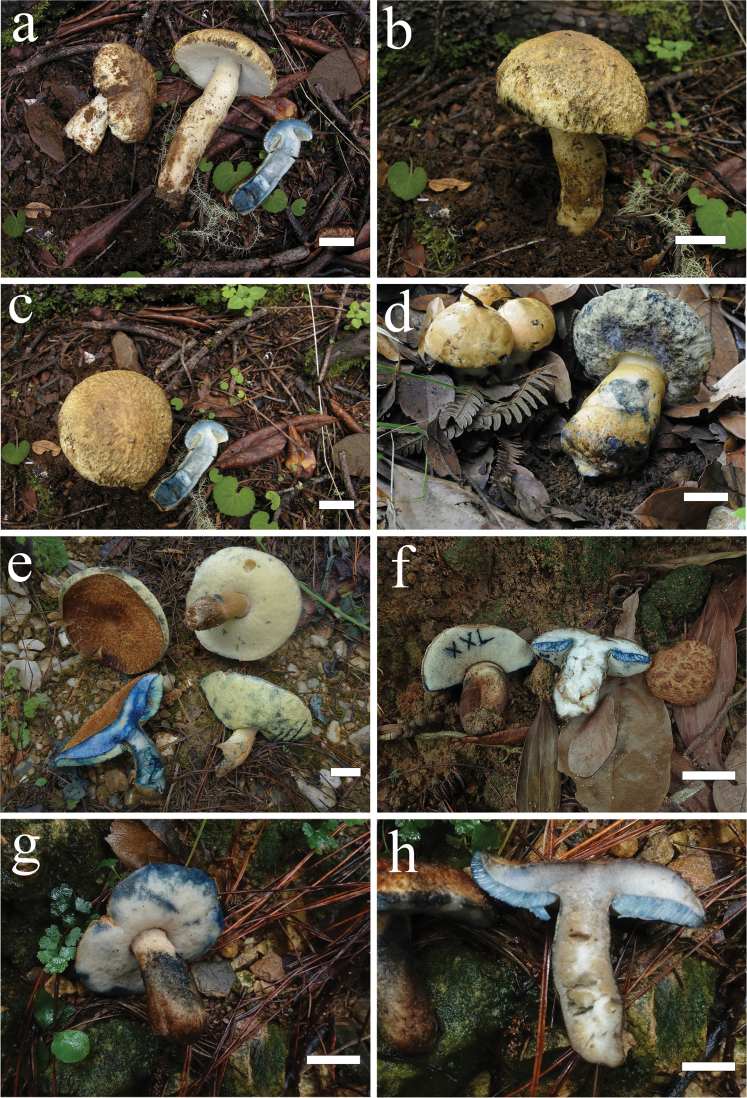
**a–c***Gyroporus alpinus* (KUN-HKAS 56318, type, photos by Y.C. Li) **d***Gyroporus flavocyanescens* (KUN-HKAS 76966, type, photo by X.L. Wu) **e–h***G. brunneofloccosus* (**e**KUN-HKAS 107735, photo by G. Wu **f**GDGM 77125, photo by J.Y. Xu **g, h**GDGM 74638, photos by J.Y. Xu). Scale bars: 2 cm.

##### Diagnosis.

This species differs from other cyanescent species of *Gyroporus* in its initially ivory yellow to greyish-yellow and then grey-orange to brownish-yellow pileus, scaly to floccose pileal surface, distribution in alpine forests with altitude up to 3800 m, broad basidiospores (5.5–8.5 µm wide) and long and slender basidia measuring 35–55 × 7–12 µm.

**Figure 4.**
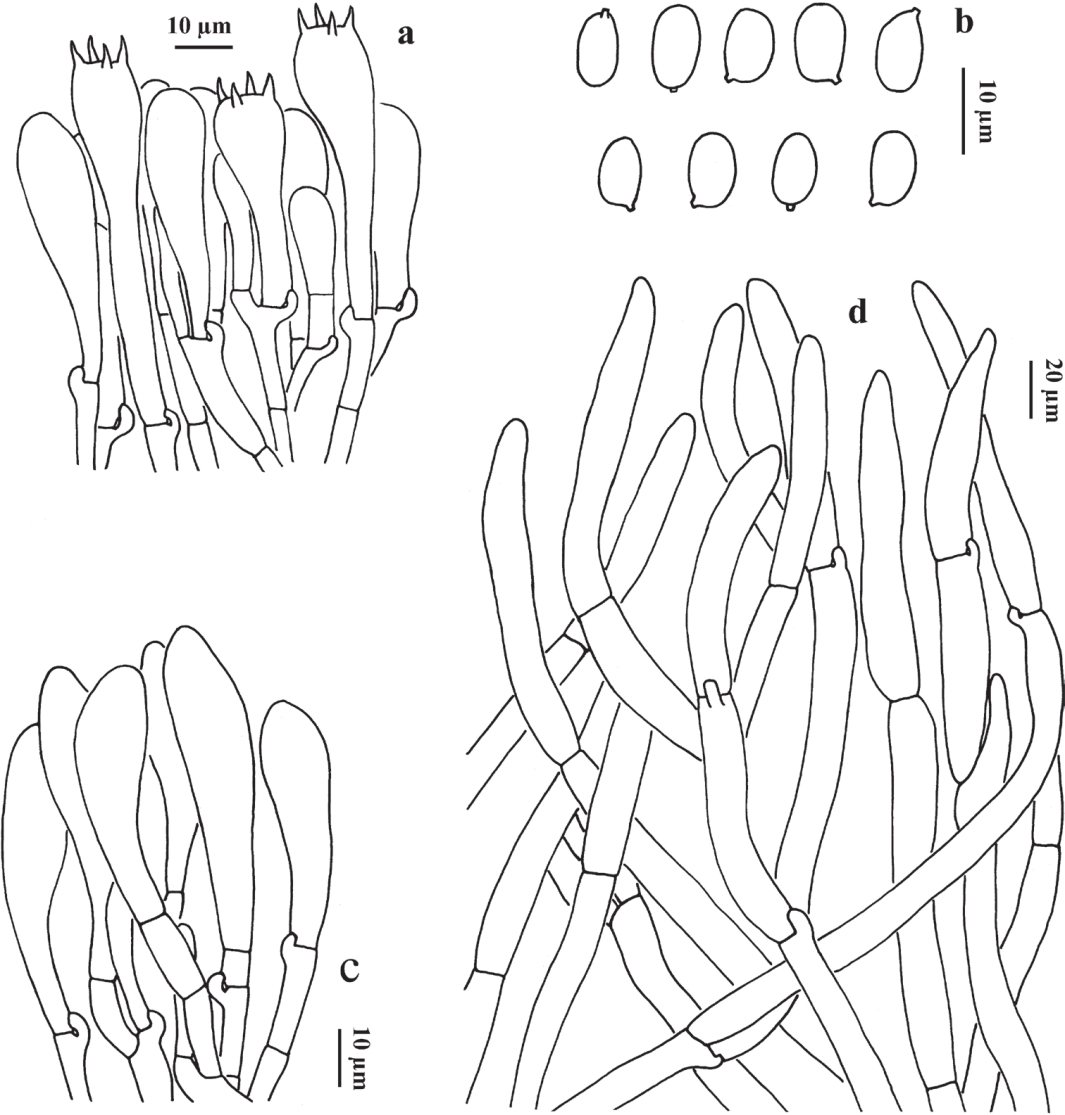
Microscopic features of *G. alpinus* (HKAS 56318, type) **a** basidia and cheilocystidium **b** basidiospores **c** cheilocystidia **d** pileipellis (squamules).

##### Description.

Pileus 3–6 cm in diam., sub-hemispherical to convex or plano-convex, ivory yellow (4B3) to greyish-yellow (2B3–4) when young, grey-orange (5B5) to brownish-yellow (5C6–7) when mature; surface dry, densely covered with concolorous appressed scaly to floccose squamules, margin always incurved and slightly extended; context whitish (1A1), staining cerulean blue (23C6–7) to dull blue (23E5–6) when bruised. Hymenophore adnate when young, slightly depressed around apex of stipe when mature; surface white (1A1) when young and then cream to yellowish when mature, staining dull blue (23E5–6) when bruised; pores angular to roundish, fine, 2–3 per mm; tubes 3–8 mm long, whitish (1A1), staining dull blue when bruised. Stipe 6–8 × 1.8–2 cm, sub-cylindrical to clavate, white (2A1) when young, yellowish-white (2A2) to concolorous with pileal surface when mature; surface roughened, staining dull blue when bruised; context white to cream or yellowish, spongy when young and then hollow in age, staining cerulean blue to dull blue when bruised. Odour indistinct and taste mild.

Basidia 35–55 × 7–12 µm, clavate, 4-spored, hyaline in potassium hydroxide (KOH) and yellowish in Melzer’s Reagent. Basidiospores [60/3/2] (6.5) 7–10 × 5.5–7.5 (8.5) µm, [Q = 1–1.65 (1.72), Q_m_ = 1.27 ± 0.23], smooth, ellipsoid to somewhat broadly ellipsoid, yellowish in KOH and primrose yellow in Melzer’s Reagent. Cheilocystidia 30–60 × 8–14 μm, clavate to subfusiform, thin-walled, hyaline in KOH and yellowish to yellow in Melzer’s Reagent; Pleurocystidia not observed. Tube trama composed of 6–11 μm wide interwoven hyphae, hyaline to yellowish in KOH, yellowish to brownish-yellow in Melzer’s Reagent. Squamules on pileus composed of 10–17 μm wide interwoven hyphae, hyaline to yellowish in KOH, yellowish to brownish-yellow in Melzer’s Reagent; terminal cells 80–120 × 12–17 μm, clavate to subcylindrical. Clamp connections frequently present in all tissues.

##### Additional specimen examined.

China. Yunnan Province: Deqin, Shangri-La County, Baima Snow Mountain, alt. 3700 m, 11 Jul 1981, L.S. Wang 827 (KUN-HKAS 7756).

##### Habitat and distribution.

Scattered on soil in alpine mixed forests dominated by *Abies* and *Picea* (Pinaceae) and *Quercus* (Fagaceae). Currently known from south-western China.

##### Note.

*Gyroporus alpinus* is characterised by the initially ivory yellow to greyish-yellow and then grey-orange to brownish-yellow pileus with scaly to floccose squamules, the slightly extended pileal margin, the white pileal context staining cerulean blue to dull blue when bruised, the white to cream or yellowish hymenophore staining dull blue when bruised, the white to yellowish-white stipe, the spongy and then hollow context in the stipe, the frequent clamp connections in all tissues, the ellipsoid to somewhat broadly ellipsoid basidiospores and the distribution in alpine forests dominated by plants of the families Pinaceae and Fagaceae. In China, specimens of *G. alpinus* have been identified as *G. cyanescens* (Ying and Zang 1994; Zang 2013). Indeed, *G. alpinus* is closely related to *G. cyanescens* (Figs 1, 2). However, *G. cyanescens*, originally described from Europe, can be distinguished from *G. alpinus* by its relatively large basidiomata which are measuring 5.1–12.7 cm in diam., pale straw-coloured pileus, relatively narrow basidiospores measuring (7) 9–11 × 4.5–6 µm and distribution in forests dominated by *Pinus sylvestris* or *Fagus sylvatia* (Fries 1821; Watling 1970; Vizzini et al. 2015).

In our analysis of the *atp6* dataset, sequences of *G. alpinus* cluster together with sequences labelled as *G. cyanescens* from South Korea and Japan without statistical support (Fig. 1). Nagasawa (2001) treated the Japanese cyanescent taxon as G. cyanescens var. violaceotinctus Watling, because of the similar colours of their basidiomata and the similar-sized basidiospores. However, G. cyanescens var. violaceotinctus, originally described from Michigan, USA, is characterised by the white to tan context staining lilaceous and then indigo when bruised, the small basidia measuring 18–23.5 × 8–9 µm, the small cheilocystidia measuring 22.5–27.5 × 4.5–7.5 µm and the distribution in forests dominated by *Acer* (Aceraceae) and *Betula* (Betulaceae) (Watling 1969). These traits are greatly different from those of *G. cyanescens* and, therefore, Blanco-Dios (2018) treated G. cyanescens var. violaceotinctus as a novel species *G. violaceotinctus* (Watling) Blanco-Dios, while the Japanese taxon differs from *G. violaceotinctus* in its white context staining greyish-blue at first and then blackish-blue when bruised without any lilaceous or violaceous tint, relatively large basidia measuring 24–42 × 9–11 µm, two types of cheilocystidia with the slender type measuring 30–64 × 6–12 µm and the voluminious type measuring 18–55 × 15–20 µm and distributions in mixed forest dominated by *Fagus* (Fagaceae), *Quercus* (Fagaceae), *Betula* (Betulaceae), *Carpinus* (Betulaceae) and *Acer* (Aceraceae) (Nagasawa 2001). The Chinese *G. alpinus* can be distinguished from *G. violaceotinctus* and the Japanese taxon by the dimensions of its basidiospores and basidia, morphology of cheilocystidia and host plants.

*Gyroporus alpinus* is phylogenetically related and morphologically similar to *G. pseudocyanescens* originally described from Spain in Crous et al. (2017) in our analysis of the combined dataset (Fig. 2). However, *G. pseudocyanescens* has a strawish-cream to yellow cream and then more or less brownish to yellowish-brown pileus, a velutinous pileal surface often cracking at maturity, relatively narrow basidiospores measuring 8–11 × 4.5–6 (6.5) µm, short terminal cells of the hyphae on the surface of the pileus measuring 50–80 × 9–15 μm and a distribution in forests dominated by *Pinus* spp. or *Quercus* spp. (Crous et al. 2017).

#### Gyroporus brunneofloccosus

Taxon classificationFungiBoletalesGyroporaceae

T.H. Li, W.Q. Deng & B. Song, Fungal Diversity 12: 123 (2013), figs 1–3

7216A3D9-0626-5C23-8494-10990CF60FDF

Figs 3e–h , 5

##### Description.

Pileus 6–9 cm in diam., hemispherical to sub-hemispherical when young, applanate to plano-convex when mature, dark brown (7E5–6) to brown (6E7–8) when young and brown to light red-brown (8E5–6) when mature; surface covered with concolorous floccose-scaly to coarsely tomentose squamules, always cracked with olivaceous yellow (2D5–6) background exposed when mature or aged, margin always extended; context white (1A1), staining cerulean blue (23C6–7) or greenish-blue (24B6–7) to dark blue (23F7–8) or deep blue (22E6–8) when bruised. Hymenophore adnate to obviously depressed around apex of stipe; surface yellowish (29B3) to pale yellow (30B3) when young and then greenish-yellow (29B5–6) when mature or aged, staining cerulean blue to greenish-blue when bruised; pores angular to roundish, 1–2 per mm; tubes 3–9 mm long, concolorous with hymenophoral surface, staining cerulean blue to greenish-blue when bruised. Stipe 4.5–6 × 1–2 cm, subcylindrical to clavate, concolorous with pileal surface when mature, but much paler when young; surface covered with tomentose to fibrillose squamules; context white to cream, spongy then hollow when mature, staining cerulean blue to greenish-blue or dark blue to deep blue when bruised. Odour and taste indistinct.

**Figure 5.**
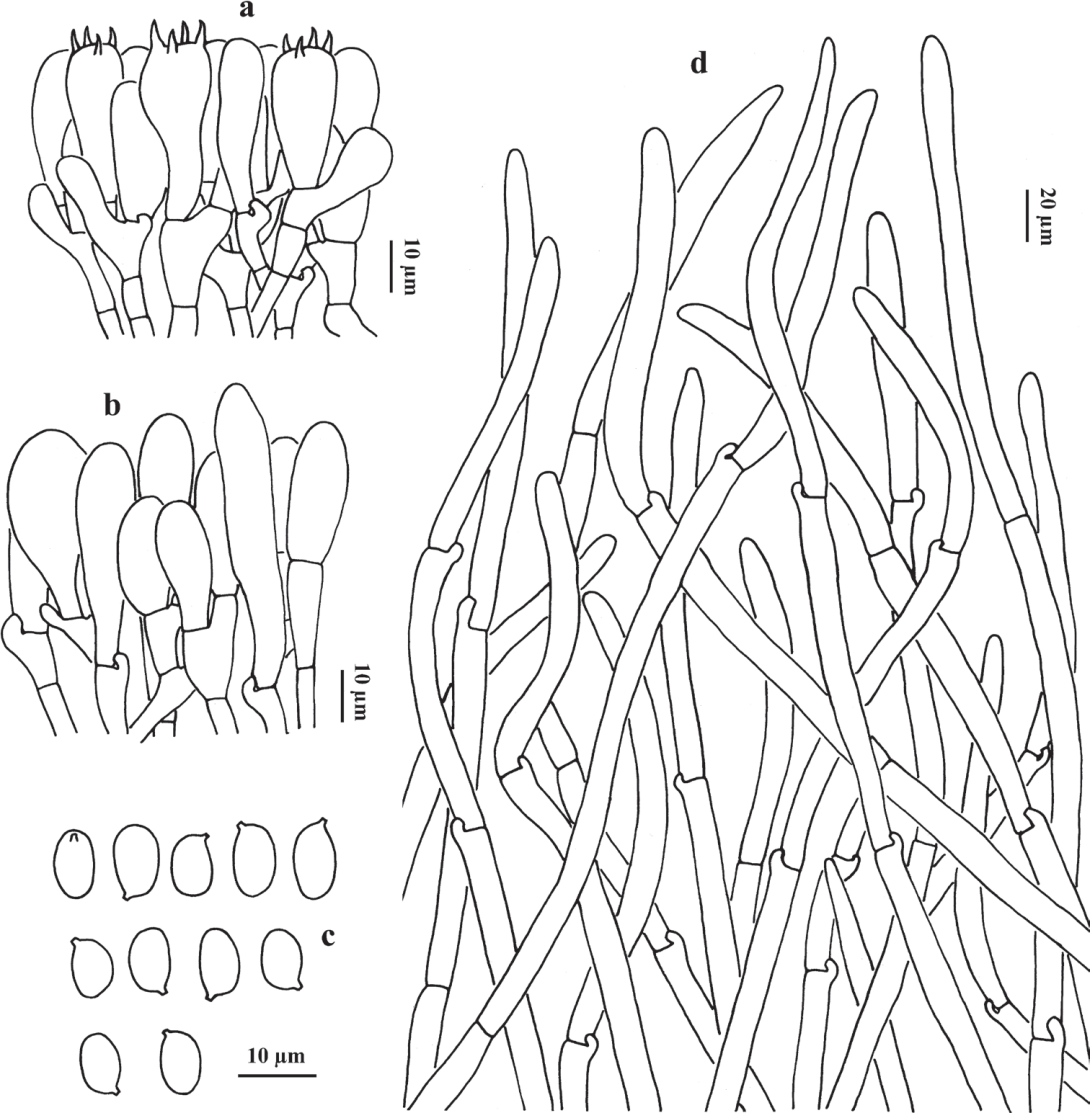
Microscopic features of *G. brunneofloccosus* (HKAS 107735) **a** basidia **b** cheilocystidia **c** basidiospores **d** pileipellis (squamules).

Basidia 22–32 × 8–11 µm, clavate, 4-spored, hyaline in KOH, yellowish in Melzer’s Reagent. Basidiospores [60/3/2] (8) 8.5–10 × 5–6 µm, (Q = 1.42 – 1.90, Q_m_ = 1.62 ± 0.11) ellipsoid, smooth, hyaline to yellowish in KOH and primrose yellow to yellowish-brown in Melzer’s Reagent. Cheilocystidia 27–44 × 9–12 μm, clavate to subfusiform, thin-walled, hyaline in KOH and yellowish in Melzer’s Reagent. Pleurocystidia not observed. Tube trama composed of 8–10 μm wide interwoven hyphae, hyaline in KOH, yellowish in Melzer’s Reagent. Squamules on pileus composed of 7–10 μm wide interwoven hyphae, hyaline to yellowish in KOH, yellowish to brownish-yellow in Melzer’s Reagent; terminal cells 80–180 × 8–10 μm, clavate to subcylindrical. Clamp connections frequently present in all tissues.

##### Specimens examined.

China. Yunnan Province: Wenshan County, Malipo Village, alt. 1200 m, 14 Oct 2017, Wu 2644 (KUN-HKAS 107735, GenBank accession numbers: MW149436 for ITS, MW151267 for nrLSU, MW452611 for *atp6*). Guangdong Province: Zhaoqing County, Dinghu Shan Nature Reserve, alt. 200 m, 28 Aug 2018, J.Y. Xu (GDGM 74638, GenBank accession numbers: MW149437 for ITS, MW151266 for nrLSU); Shenzhen, Songzikeng Forest Park, alt. 70 m, 19 Jul 2019, J.Y. Xu (GDGM 77125).

##### Habitat and distribution.

Scattered on soil in tropical forests dominated by *Castanopsis* (Fagaceae), *Quercus* (Fagaceae) and *Pinus* (Pinaceae). Currently known from southern and south-western China.

##### Discussion.

*Gyroporus brunneofloccosus*, originally described from southern China, is characterised by the initially dark brown to brown and then brown to light red-brown pileus with concolorous floccose-scaly to coarsely tomentose squamules, the extended pileal margin, the white pileal context staining cerulean blue or greenish-blue to dark blue or deep blue when bruised, the initially yellowish to pale yellow and then greenish-yellow hymenophore staining cerulean blue to greenish-blue when bruised, the brownish to brown or light red-brown stipe, the spongy and then hollow context in the stipe, the frequent clamp connections in all tissues, the ellipsoid basidiospores and the distribution in tropical forests dominated by plants of the families Fagaceae and Pinaceae (Li et al. 2003).

In China, *G. brunneofloccosus* was misidentified as *G. cyanescens* by Bi et al. (1990, 1994), Ying and Zang (1994) and Mao (2000). However, these two species can be separated both by phylogenetic and morphological evidence. Our phylogenetic analysis of *atp6* data (Fig. 1) indicates that *G. brunneofloccosus* is closely related to *G. phaeocyanescens*. However, *G. phaeocyanescens*, originally described from Belize by Singer et al. (1983), has fulvous to snuff brown pileus and relatively large basidiospores measuring 9.3–14.7 × 5.3–6.7 µm (Singer et al. 1983; Li et al. 2003).

#### Gyroporus flavocyanescens

Taxon classificationFungiBoletalesGyroporaceae

Yan C. Li, C. Huang & Zhu L. Yang sp. nov.

AFFCE15D-376D-583F-AE77-94F33CE2A21B

838414

Figs 3d , 6

##### Etymology.

The epithet *flavocyanescens* refers to the flavous basidiomata with blue discolouration when bruised.

**Figure 6.**
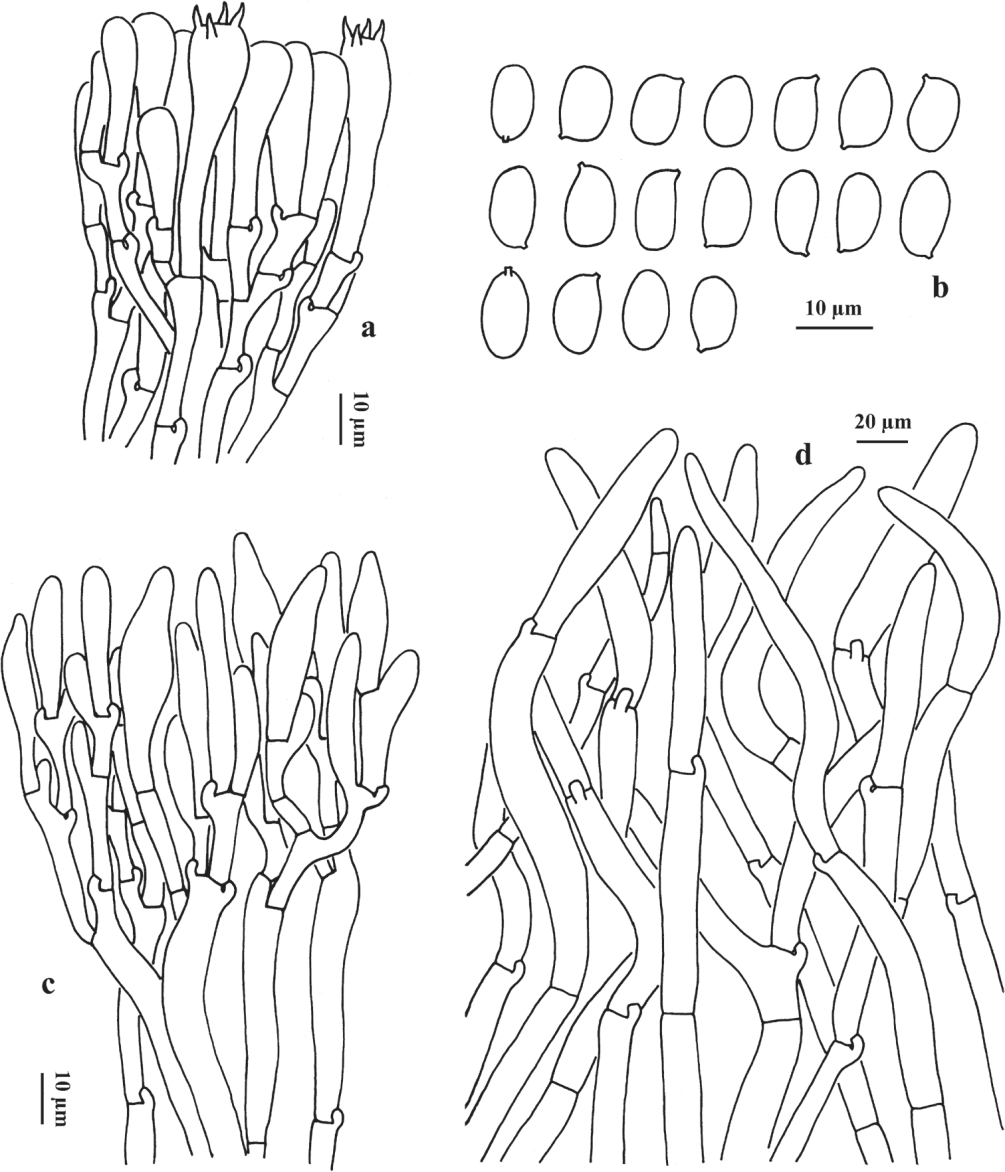
Microscopic features of *G. flavocyanescens* (KUN-HKAS 76966, type) **a** basidia **b** basidiospores **c** cheilocystidia **d** pileipellis (squamules).

##### Type.

China. Guizhou Province: Pan County, alt. 1700 m, 2 Jul 2008, X.L. Wu 1182 (KUN-HKAS 76966, GenBank accession numbers: MW440550 for ITS, MW442950 for nrLSU, MW452613 for *atp6*).

##### Diagnosis.

Differs from other cyanescent species in *Gyroporus* by its initially flavous to dull yellow or grey-yellow and then grey-orange to greyish-orange pileus, nearly glabrous or somewhat fibrillose to finely tomentose pileal surface, relatively small basidia measuring 21–30 × 9–11 µm, slender basidiospores measuring 8–10 × (5) 5.5–6.5 µm and relatively short and slender chelocystidia measuring 26–35 × 5–9 μm.

##### Description.

Pileus 6–10 cm in diam., hemispherical to sub-hemispherical when young, applanate to plano-convex when mature, flavous (3B3–4) to dull yellow or grey-yellow (2B4–5) when young, grey-orange (5B4–5) to greyish-orange (5B3–4) when mature; surface dry, nearly glabrous or somewhat fibrillose to finely tomentose, margin incurved and slightly extended; context whitish (1A1), staining strong dark blue or indigo-blue (24D4–8) when bruised. Hymenophore adnate when young, depressed around apex of stipe when mature; surface white (1A1) when young and then grey (1B1) to cream when mature, staining cyanine blue (24D4–6) to porcelain blue (23C5–6) when bruised; pores angular to roundish, 1–2 per mm; tubes 4–10 mm long, whitish (1A1), staining cyanine blue to porcelain blue when bruised. Stipe 4–6 × 2.5–4 cm, clavate, enlarged downwards; surface roughened, white to cream when young and then pale yellow (2A3–5) to concolorous with pileal surface when mature or aged; context white to cream or yellowish, spongy when young and then hollow in age, staining cyanine blue to porcelain blue when bruised. Odour indistinct and taste mild.

Basidia 21–30 × 9–11 µm, clavate, hyaline in KOH and yellowish in Melzer’s Reagent, 4-spored. Basidiospores [60/3/2] 8–10 × (5) 5.5–6.5 µm, (Q = 1.45–1.81, Q_m_ = 1.59 ± 0.12), smooth, ellipsoid to somewhat broadly ellipsoid, hyaline to yellowish in KOH and primrose yellow to yellow in Melzer’s Reagent. Cheilocystidia 26–35 × 5–9 μm, clavate to subfusiform, thin-walled, hyaline in KOH and yellowish to yellow in Melzer’s Reagent. Pleurocystidia not observed. Tube trama composed of 5–9 μm wide interwoven hyphae, hyaline to yellowish in KOH, yellowish to brownish-yellow in Melzer’s Reagent. Squamules on pileus composed of 8–17 μm wide interwoven hyphae, hyaline to yellowish in KOH, yellowish to brownish-yellow in Melzer’s Reagent; terminal cells 90–140 × 9–17 μm, clavate to subcylindrical. Clamp connections frequently present in all tissues.

##### Additional specimen examined.

China. Guizhou Province: Pan County, alt. 1700 m, 2 Jul 2008, X.L. Wu 1187 (KUN-HKAS 77804, GenBank accession numbers: MW440551 for ITS, MW442951 for nrLSU).

##### Habitat and distribution.

Scattered on soil in the tropical forests dominated by *Castanea* sp. (Fagaceae) and *Quercus* sp. (Fagaceae). Currently known from south-western China.

##### Note.

*Gyroporus flavocyanescens* is characterised by the flavous to dull yellow or grey-yellow and then grey-orange to greyish-orange pileus, the nearly glabrous to fibrillose to finely tomentose pileal surface, the slightly extended pileal margin, the white pileal context staining strong dark blue or indigo-blue when bruised, the white to grey or cream to yellowish hymenophore staining cyanine blue to porcelain blue when bruised, the white to cream and then pale yellow to flavous stipe, the spongy and then hollow context in the stipe, the frequent clamp connections in all tissues, the ellipsoid to somewhat broadly ellipsoid basidiospores and the distribution in tropical forests dominated by plants of the family Fagaceae.

*Gyroporus flavocyanescens* is morphologically similar to *G. lacteus* and *G. pseudolacteus*. Indeed, they are phylogenetically related to each other, based on our analysis of combined nrLSU + ITS dataset (Fig. 2), though it should be noted that the bootstrap support is relatively low for the relationship with *G. lacteus* (87%). *Gyroporus lacteus* has large basidiomata (9–17 cm in diam.), ochraceous pileus with scaly tomentose squamules and large cheilocystidia up to 50 × 10 µm (Vizzini et al. 2015). *Gyroporus pseudolacteus* has a whitish to cream white and then more or less yellowish-ochre pileus, relatively large basidia measuring 35–43 × 10–14 µm and large cheilocystidia measuring 35–55 × 8–12 µm (Crous et al. 2017).

In this study, three cyanescent species of *Gyroporus* from China could be recognised and identified. For the convenience of identification of the species, a key is given below.

### Key to cyanescent *Gyroporus* species in China

**Table:** 

1	Pileus dark brown, brown to light red-brown, without any yellow or orange tinge; squamules on pileus composed of 7–10 μm wide interwoven hyphae	***G. brunneofloccosus***
–	Pileus ivory yellow to greyish-yellow or flavous to grey-yellow and then grey-orange to brownish-yellow, without brown tinge; squamules on pileus composed of broad interwoven hyphae up to 17 μm wide	**2**
2	Basidioma distributed in alpine mixed forests dominated by *Abies* sp., *Picea* sp. and *Quercus semicarpifolia*; pileus small to medium-sized 3–6 cm wide, ivory yellow to greyish-yellow and then grey-orange to brownish-yellow, surface with scaly to floccose squamules; cheilocystidia 30–60 × 8–14 μm, clavate to subfusiform; basidia 35–55 × 7–11.5 μm	***G. alpinus***
–	Basidioma distributed in tropical forests dominated by *Castanea* sp. and *Quercus* sp.; pileus large 6–10 cm wide, flavous to dull yellow or grey-yellow and then grey-orange to greyish-orange, surface nearly glabrous or somewhat fibrillose to finely tomentose; cheilocystidia relatively small measuring 26–35 × 5–9 μm; basidia relatively short measuring 21–30 × 9–11 μm	***G. flavocyanescens***

## Discussion

Cyanescent *Gyroporus* species in the Southern Hemisphere form independent lineages in the analyses of *atp6* and combined nrLSU + ITS datasets (Figs 1, 2) and mainly associate with plants of the family Myrtaceae, while the cyanescent species in the Northern Hemisphere also form independent lineages, but without or with low statistical bootstrap support in the analyses of the combined and *atp6* datasets and mainly associate with plants of the families Fagaceae and Pinaceae. Davoodian et al. (2020) suggest that the Southern Hemisphere lineage is derived from the Northern Hemisphere lineage, within which the Southern Hemisphere lineage is embedded. Further field investigations, careful morphological observations and extensive molecular analysis using multiple genes should help better understand the geographical relationships amongst the cyanescent species.

Sixteen cyanescent *Gyroporus* species were revealed, based on former and present studies, including nine distributed in the Northern Hemisphere and seven distributed in the Southern Hemisphere. Three cyanescent *Gyroporus* have been reported from China before our study, namely *G. cyanescens*, *G. brunneofloccosus* and *G. pseudomicrosporus* (Zang 1986, 2013; Bi et al. 1990, 1994; Ying and Zang 1994; Mao 2000; Li et al. 2003). *Gyroporus cyanescens* was regarded as geographically widespread in Europe, North America and East Asia in the past. Our study identified the disjunct populations of this taxon in Europe and North America, but its distribution in China has not been found yet. The specimens from China labelled “*G. cyanescens*” are either *G. alpinus* or *G. flavocyanescens*. *Gyroporus pseudomicrosporus*, originally described from China by Zang (1986), is characterised by the cyanescent discolouration when bruised, the decurrent hymenophore, the short tubes measuring 2–4 mm long, the eccentric stipe and the small ellipsoid to ovoid basidiospores. These traits match well with those of the genus *Gyrodon* Opat. In conclusion, there are still three cyanescent species in China, but they are *G. alpinus*, *G. brunneofloccosus* and *G. flavocyanescens*.

## Supplementary Material

XML Treatment for Gyroporus alpinus

XML Treatment for Gyroporus brunneofloccosus

XML Treatment for Gyroporus flavocyanescens
